# Force-enhanced biophysical connectivity of platelet β3 integrin signaling through Talin is predicted by steered molecular dynamics simulations

**DOI:** 10.1038/s41598-022-08554-w

**Published:** 2022-03-17

**Authors:** Shuixiu Su, Yingchen Ling, Ying Fang, Jianhua Wu

**Affiliations:** Institute of Biomechanics/School of Biology and Biological Engineering, South China University of Technology, Guangzhou, 510006 China

**Keywords:** Cellular signalling networks, Molecular modelling

## Abstract

Platelet β3-integrin signaling through Talin is crucial in platelet transmembrane signaling, activation, adhesion, spreading and aggregation, and remains unclear in mechano-microenvironments. In order to examine Talin-β3 integrin biophysical connectivity, a series of “ramp-clamp” steered molecular dynamics (SMD) simulations were performed on complex of F3 domain of Talin and cytoplasmic tail of β3 integrin to imitate different force-loads in platelet. Pull-induced allostery of the hydrophobic pocket in F3 domain might markedly enhance complex rupture-force (> 150pN) and slow down breakage of the complex; the complex should mechano-stable for its conformational conservation under loads (≤ 80pN); increasing force below 60pN would decrease the complex dissociation probability, and force-induced extension of β5 strand on Talin and binding site residues, ASP^740^ and ALA^742^ as well as Asn^744^, on β3-integrin were responsible for the force-enhanced linkage of the Talin-β3 integrin. Force might enhance biophysical connectivity of β3-integrin signaling through Talin by a catch bond mechanism, which be mediated by the force-induced allostery of complex at clamped stage. This work provides a novel insight into the force-regulated transmembrane β3-integrin signaling and its molecular basis for platelet activation, and exhibited a potential power of the present computer strategy in predicting mechanical regulation on ligand-receptor interaction under loads.

## Introduction

Integrins, a family of transmembrane glycoprotein receptor, are comprised of two noncovalently associated subunits, α and β, and play essential role in tissue organization, immune responses, leukocyte traffic and cell development by linking the extracellular matrix to intracellular signaling pathway^1^. As a predominant type of integrin only expressed on platelet surface, α_IIb_β_3_, is necessary for platelet functions, hemostasis and thrombosis^2^. Under physiological condition, β3 integrin on circulating platelet is at an inactive bend headpiece-closed conformation usually and cannot bind their ligands^3^. At vascular injury site, α_IIb_β_3_ integrin can be induced to an active state through subendothelial von Willebrand factor (vWF) and virous platelet agonists, such as adenosine diphosphate (ADP), platelet-activating factor (PAF) and thrombin^4–7^. Interaction of activated α_IIb_β_3_ with its major ligand such as fibrinogen mediates platelet adhesion, spreading and aggregation as well as thrombus growth^1,5,8^. Defect in integrin expression or function causes various diseases of immune and bleeding disorder, such as Glanzmann thrombasthenia and leukocyte adhesion deficiencies^2,9,10^.

The short cytoplasmic tail (CT) of β3 integrin provides multiple binding sites for adaptors and signaling proteins, which are critical in bidirectional integrin signaling^11,12^. In initial activation of β3 integrin via a variety of “inside-out” signaling, the final step is Talin binding to integrin CT with or without kindlin assistance^13–16^. Talin, a cytoplasmic protein, consists of an atypical FERM domain (F0–F1–F2–F3) that contains a high affinity binding site for β integrin CT, a large unstructured link region and a flexible rod domain that contains multiple binding sites for actin, vinculin, Ras-related protein 1 (Rap1) and Rap1-GTP-interacting adaptor molecule (RIAM)^17^. Talin ligated to the first NPxY motif on integrin β3 tail triggers an unclasping of intracellular and transmembrane domains between αIIb and β3 subunits, inducing extracellular αIIbβ3 conformation change and ligand-binding affinity promotion^5,8,12,13^. After ligand binding, αIIbβ3 undergoes a further ligand-induced conformational change and initiates a cascade of intracellular signaling events^18^. In this “outside-in” signaling, Talin, as a key player, couples β3 integrin CT to cytoskeletal actin, and this dynamic molecular clutch formation is critical for platelets to sense and respond to microenvironmental signals^16,19–21^.

**Figure 1:**
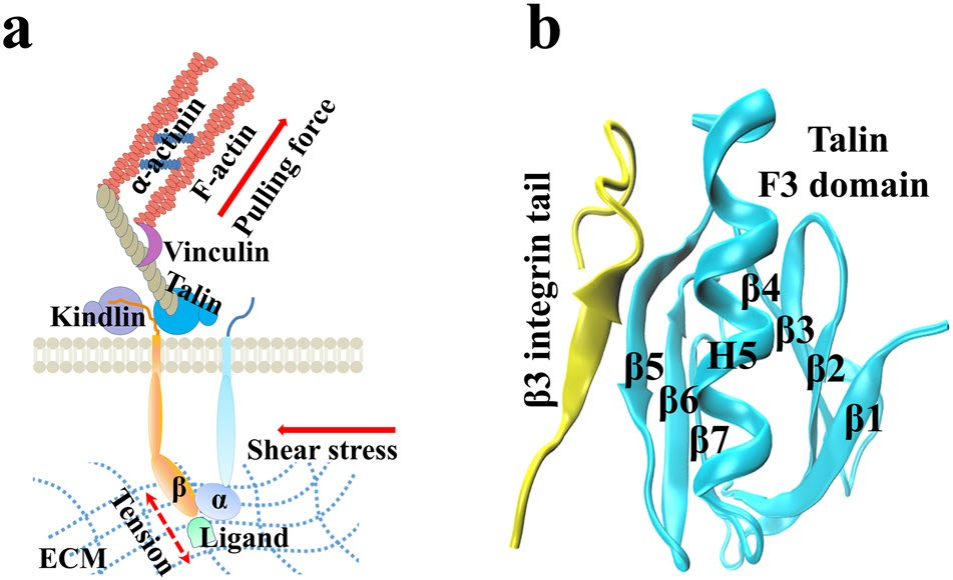
The complex of Talin F3 domain and cytoplasmic distal (CD)-membrane region of β3 integrin in signaling. (**a**) Schematic diagram of transmembrane β3 integrin signaling through Talin under mechanical micro-environment. (**b**) The crystal structure of Talin head F3 domain bound with β3 integrin CD domain. Talin F3 domain consists of two antiparallel β sheets composed of four and three strands, packed into a β sandwich enclosing a hydrophobic core, with a single α helix at the C terminus packing between β5 and β1, partly enclosing one edge of the sandwich. The integrin β3 tail includes a small sheet in the middle and two flexible loops at both terminals.

Both mechanosensitive integrin and Talin are capable of altering their ligand-binding affinities through force-induced conformational changes^22–24^. An axial traction and a lateral resistance from extracellular matrix can be sensed and transmitted highly by bonds between integrins and their ligand^5,25^. Increasing force within a certain range makes bond lifetime prolonged, suggesting a catch bond^18,24,26^, which mediates stable platelet adhesion on injury vessel site and arterial thrombus formation at high pathological shear stress^5,27,28^. It is from the quantitative evaluation results of several existing integrin activation models that tensile force of 1–3 pN from cytoskeleton is a more potent regulator than physiological Talin concentration in maintaining rare active conformation of integrin^23,29^. And, unfolding of Talin rod domain and switching from RIAM to vinculin-ligated Talin are tightly regulated by mechanical force, which is crucial during adhesion assembly and maturation^22,30^. Therefore, it is inferred that, as a bridge between the extracellular ligand and the intracellular actin, the Talin-integrin linkage must withstand forces from both of extracellular blood shear stress and cytoskeletal rearrangement in platelet spreading and aggregation (Fig. 1a).

However, regulation of force on physical connectivity of the Talin-integrin linkage remains unclear. Less knowledge is about both Talin affinity to β3 integrin and its mechano-regulation mechanism also. Previous studies focused on force-dependent stochastic unfolding and refolding of Talin rod domains^31,32^ but not the interaction of β3 integrin to Talin head domain, despite that crystal structure of Talin bound with the β3-integrin CT domain had been solved^33^ (Fig. 1b). To reveal the mechanical regulation on biophysical connectivity of β3-integrin signaling through Talin and its molecular basis, we herein investigated interaction of β3-integrin CT domain to Talin F3 domain under various mechanical loads through a series of molecular dynamics (MD) simulations, which were performed for modelling molecular interactions successfully in various extracellular and intracellular molecular systems^34–36^. The present results revealed a force-enhanced biophysical connectivity of β3 integrin signaling through Talin, its mechano-kinetics regulation mechanism and structural basis, provide not only a novel insight into β3 integrin signaling in platelet activation, hemostasis and thrombosis under mechano- microenvironments but also a new clue for drug design and treatment of thrombotic diseases.

## Results

### The dominant interactions of binding site residues in equilibrated complex of Talin F3 domain with CT domain of β3 integrin

**Figure 2:**
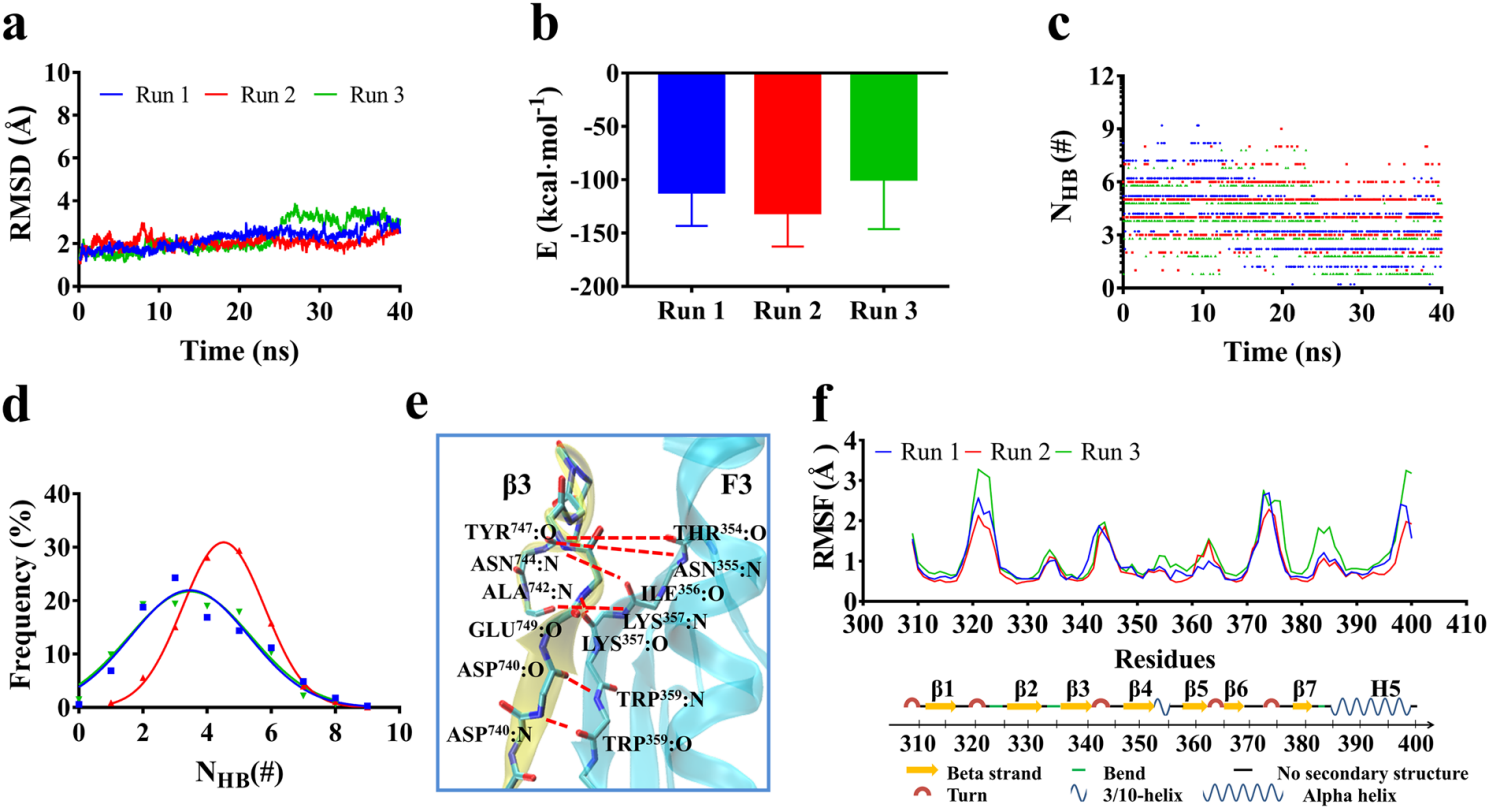
The dominant interactions of binding site residues in equilibrated complex of Talin F3 domain with CT domain of β3 integrin. (**a**) The time courses of Cα-RMSD, (**b**) the mean interaction energy over 40 ns for the equilibrated complexes, (**c**) the time course of interface H-bond number (N_HB_) and (**d**) the N_HB_ distribution with 40 ns for three Runs. The Cα-RMSD was limited in region from 1 to 4 Å, showing the system at equilibrium. The best fitting results of N_HB_ frequency data showed a Gaussian distribution, and the maximum N_HB_ and the minimum E were obtained in Run2, meaning that the equilibrated complex from Run2 was the best for subsequent simulations. Interaction between binding site residues of complex and RMSF pattern of residues on the ligated Talin F3 domain. (**e**) The key interface H-bonds (red dotted line) and their respective involved binding site residue pairs in complex of β3 integrin tail (yellow) with Talin F3 domain (cyan) in F3 domain. (**f**) The Cα-RMSF patterns of residues on equilibrated Talin F3 domains respectively for Run1 (blue), Run2 (red) and Run3 (green).

To uncover the dominant binding site residues in binding of β3 integrin CT domain to Talin F3 domain at physiological conditions, three equilibrated structures of the complex were obtained herein by performing 40 ns system equilibrium thrice (Materials and Methods), along a same protocol of energy minimization and hypothesis that the complex was equilibrated if the time courses of the RMSD of heavy atoms, temperature and total energy were fluctuated on their respective stable levels with small relative derivations (Fig. 2a). In the three equilibrated structures, one from Run2 should be the most stable, because the mean number (N_HB_) of interfacial H-bonds was about 4.3 for the structure (with mean interaction energy of −135 kcal·mol^-1^) from Run2 but 3.7 for others (with mean interaction energies larger than −120 kcal·mol^-1^) from Run1 and Run3 (Fig. 2b-d). Numbered one by one as time passed through, the interface H-bonds obeyed a Gaussian distribution with R^2^ > 0.98 if the conformations were sampled within simulation times over 40 ns (Fig. 2c, d), suggesting that the conformation space of the complex sampled herein in 40 ns equilibrium should be quasi-complete.

The detected interface H-bonds in top six of occupancies were contributed by ASP^740^ with TRP^359^, TYR^747^ with ASN^355^, ASN^744^ with ILE^354^ and THR^356^, and LYS^357^ with ALA^742^ and GLU^749^ (Fig. 2e; Table 1). It could be seen that the binding sites of Talin is located on the 3/10 helix-loop-β5 strand behind β4 strand (Fig. 2e, f). Talin mutation on TRP^359^ would impair Talin binding with integrin in platelets through decelerating αIIbβ3 activation, and the critical roles of ASN^744^ and TYR^747^ on β3 integrin first ^744^NPxY^747^ motif had been demonstrated by mutation-induced disruption of Talin binding to β1 and β3 integrin^37,38^. It meant that the binding site residues, such as THR^354^, ASN^355^ and THR^356^ on Talin as well as ASP^740^ on β3 integrin, together with their respective partners, were dominant for binding of Talin F3 domain to the CT of β3 integrin. In addition to ^744^NPxY^747^ motif, the residue ALA^742^ contributed a H-bond with a substantial occupancy, but did not the residue GLU^749^, suggesting ALA^742^ rather than GLU^749^ was another key binding site residue on β3 integrin (Table 1). And, the better stability of the complex conformation from Run2 might come from the stronger interface H-bonding events, which turned complex flexibility down, especially in the link between β1 and β2 stands, the loop and front part of H5 helix behind β7, by comparing with other two conformation from Run1 and Run3 (Fig. 2e, f; Table 1). Thus, the equilibrated conformation from Run2 was regarded as the more wild-like and rational one among the three equilibrated structures, and was chosen as the initial conformation for the subsequent SMD simulations herein.

**Table 1:** Interface H-bonds between binding site residue pairs of three equilibrated complexes.

No	β3 integrin	F3 domain	Occupancy (%)			Average (%)
			Run 1	Run 2	Run 3	
1	ASP^740^	TRP^359^	97.1	97.1	91.8	95.3 ± 3.10
2	ALA^742^	LYS^357^	46.3	71.9	5.4	41.2 ± 33.5
3	ASN^744^	ILE^356^	39.3	72.2	27.0	46.2 ± 23.4
4	ASN^744^	THR^354^	20.1	53.9	0.0	24.7 ± 23.9
5	TYR^747^	ASN^355^	28.1	13.1	18.5	19.9 ± 7.60
6	GLU^749^	LYS^357^	6.0	17.9	0.0	8.00 ± 8.40

The H-bond occupancies in column 4–6 showed a mean over 40 ns simulation time of a run. Data from three runs were averaged and shown in means ± S.D. (column 7).

### Two pull-induced dissociation pathways of Talin away from β3 integrin with different intrinsic mechanical strengths

**Figure 3:**
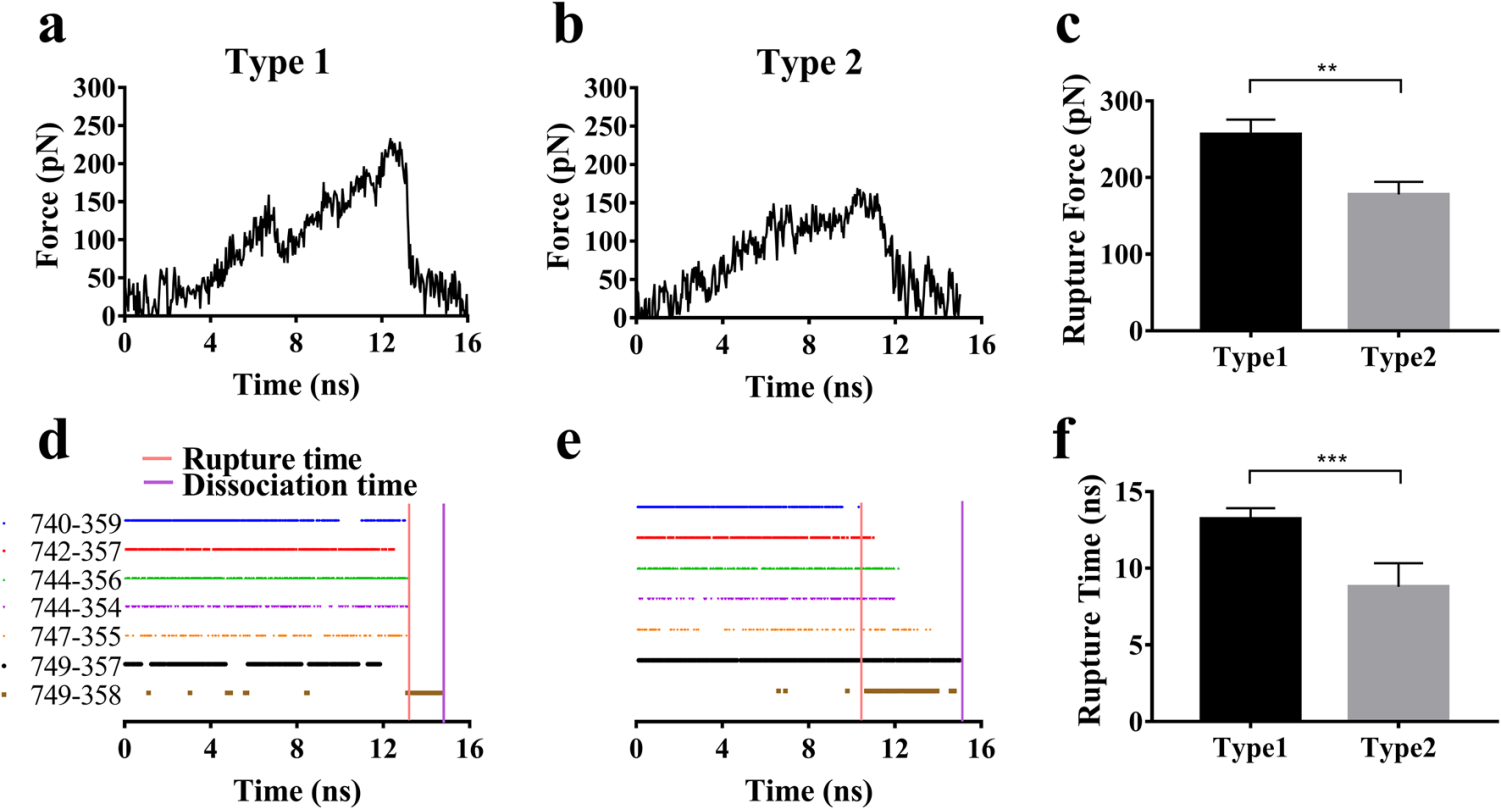
Time-courses of loading force, survival pattern of interfacial H-bonds, and rupture forces for the complex pulled with velocity of 3 Å/ns. (**a**) and (**b**) The time-curves of loading force on the complex in type 1 and 2. The force–time curves in type 1 and 2 exhibited two different representative dissociation pathways at pull velocity of 3 Å/ns. (**c**) The rupture forces during three runs in the two type dissociation pathways. (**d**) and (**e**) The survival patterns of interfacial H-bonds for the pull-induced complex dissociations in type 1(**d**) and 2(**e**). In each pattern, the unbroken and broken state of the bond were expressed by the colored and uncolored line, respectively. The pathway diversity of the pull-induce dissociation was shown in the different survival pattens of the seven involve H-bonding events. (**f**) the rupture time during three runs in the two type dissociation pathways, and another two runs of each type were presented in the Supplementary materials (Fig. S1).

To prevent from mechanical damage of β3-integrin signaling through Talin, a high mechanical strength of Talin-β3 integrin complex might be necessary. We examined the rupture force of the complex by performing 16 ns force-ramp SMD simulation thrice with time step of 2 fs and a pulling velocity of 3 Å/ns (Materials and Methods). Here, the N-terminal C_α_ atom of β3-integrin CT domain was chosen as the fixed point because of the restriction of cell membrane, while the N-terminal C_α_ atom of Talin F3 domain was selected as the stretching point to simulate the tension from the connecting skeleton actin. From total six simulations (Fig. 3a, b; Fig. S1), two representative force–time curves (Fig. 3a, b) with their respective rupture forces of 170 and 240 pN about showed a diversity of pull-induced β3-integrin dissociation from Talin (Fig. 3c). These six pull-induced dissociation pathways (Fig. 3a, b; Fig. S1) were clustered two types according to whether the rupture force was greater than 200 pN or not. In the 1st type pull-induced dissociation pathway (Fig. 3a), stretching force on the complex raised in vibration to its minor peak of about 150 pN at pull time of about 7 ns, then dropped to 80 pN about, and further increased to the main peak of 240 pN at pull time of 13 ns; and along the 2nd type pull-induced dissociation pathway (Fig. 3b), the tensile force on the complex climbed to a plateau of 150pN about at pull time of 7 ns, then fluctuated around this plateau for 3 ns, and climbed again to the main peak of 170 pN. The significant difference in the two respective rupture forces of 170 and 240pN and rupture time of 10 and 13 ns suggested a pathway-dependent pull-induced breakage of the complex (Fig. 3c-f), and demonstrated that there existed at least two pull-induced dissociation pathways.

Under pulling, the H-bonds among the six residue pairs mentioned above (Table 1) had remained stable until tensile on the complex increased to 150pN (Fig. 3d, e). The anticipatory breakage of H-bond between ASP^740^ and TRP^359^ in the 2nd type pull-induced dissociation pathway resulted in the subsequent hydrogen bonds broken like zippers, which might reduce the rupture force of the complex significantly, in comparison with the 1st type pathway (Fig. 3). It meant that the most resistance to stretching were shared by the six H-bonds mentioned above, but the newly formed but weak H-bond between GLU^749^ and ARG^358^ should play very slight role in the pull-induced β3 dissociation from Talin (Fig. 3d, e).

### Pull-induced allostery enhanced mechanical strength of Talin-β3 integrin complex

**Figure 4:**
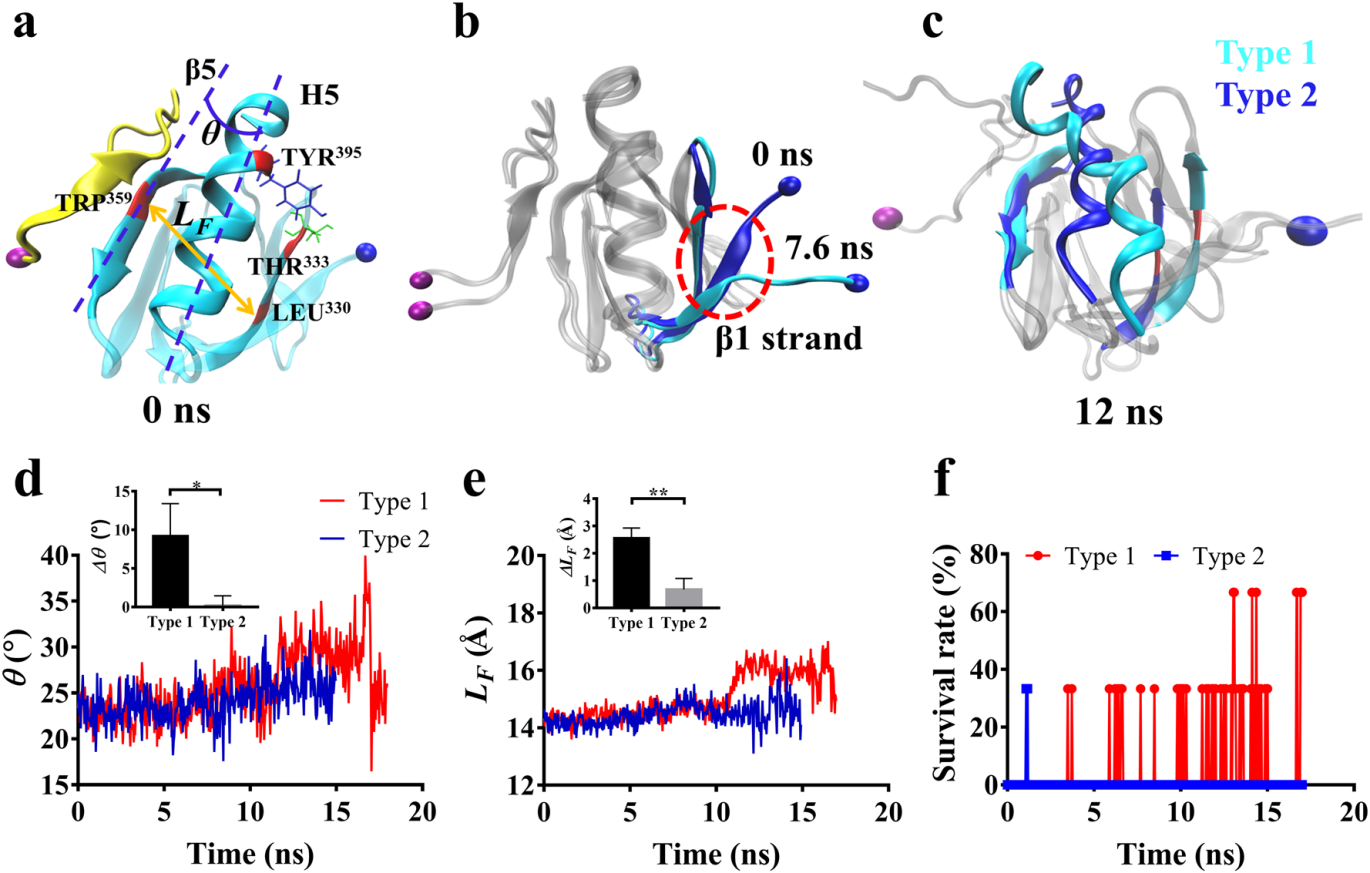
Pull-induced allostery of the complex. (**a**–**c**) The snapshots of the pulled complex of β3 integrin (yellow) with Talin F3 domain (cyan) along the dissociation pathway in type 1 at different pull time. The purple sphere was the fixed atom, and the blue sphere denoted the steered atom. (**a**) Schematic diagrams for the angle *θ* between H5 helix and β5 strand of F3 domain (blue dotted line), the distance *L*_*F*_ (cyan) between two Cα-atoms of LEU^330^ on β2-strand and TRP^359^ on β5-strand of F3 (orange line) and the newly formed H-bond between TYR^395^ on H5 helix and THR^333^ on β2-strand of F3 domain. (**b**) The β1 strand of the ligated F3 domain (cyan) became unfolded or spread at pull time of 7.6 ns or pull force of 150pN in comparison with its static state (blue) (pull time = 0 ns), and (**c**) the hydrophobic pocket of the ligated F3 domain became open at pull time of 12 ns. The time course of the angle *θ* (**d**) and the distance *L*_*F*_ (**e**) and their differences between start point and rupture point *Δθ* and *ΔL*_*F*_ in type 1 and 2 run modes, respectively. (**f**) The mean survival rate for the newly formed H-bond between TYR^395^ on H5 helix and THR^333^ on β2-strand of F3 domain in type 1 and 2 run modes, all data from three runs.

Once the H-bond between ASP^740^ and TRP^359^ broke, the binding interface area of Talin-β3 integrin complex decreased suddenly at about 13 ns in 1st type pathway, but gradually at about 10 ns in 2nd type one (Fig. S2), as shown by their time courses of force, respectively (Fig. 3a, b; Video. S1). In order to further explore the difference between these two pull-induced dissociation pathways, the conformational evolution of the complex, especially for Talin-F3 domain rather than small β3 integrin tail, was observed carefully. In both of 1st and 2nd type dissociated pathways, we found that, the conformation of ligated F3-domain remained mechano-stable for the first 7.6 ns with tensile force ≤ 120pN, then the C-terminal β1 strand began unfolding from the body as tensile force increased to 150pN about (Figs. 3, 4a, b; Video. S1). Furthermore, along the force-loading ramp, pull-induced complex allostery occurred just in the 1st type but not the 2nd type dissociation pathway (Fig. 4). This pull-induced allostery of the ligated Talin was an early event of β3-integrin dissociation from Talin (Figs. 3 and 4), and could be illustrated by that the angle *θ* between H5 helix and β5-strand increased from 22° to 30° at pull time of 12 ns, so did (from 14 to 17 Å) the distance from TRP^359^ on β5 strand to LEU^330^ on β2 strand (Fig. 4a, c, d, e), while the complex dissociation occurred at pull time of about 15 ns (Fig. 3d, e).

Through the analysis of the internal interaction network of Talin-F3 domain, it was found that pull-induced formation of new H-bond between TYR^395^ on H5 helix and THR^333^ on β2-strand might facilitate deformation and extension of the hydrophobic core (Fig. 4a, f), which partly enclosed by H5 helix packing between β5 and β1 (Fig. 1b). Due to TYR^395^ - THR^333^ H-bond emersion, the enhanced interaction between H5 helix and β2-strand would promote H5 helix swing outward with β2-strand along the pull direction, and further made exposure of hydrophobic pocket in F3-domain easy. The allostery-induced increasing in rupture-time and rupture-force might be required in preventing the complex from mechanical damage especially under pathological high shear stresses.

### Biphasic regulation of force on interaction of β3 integrin with Talin

**Figure 5:**
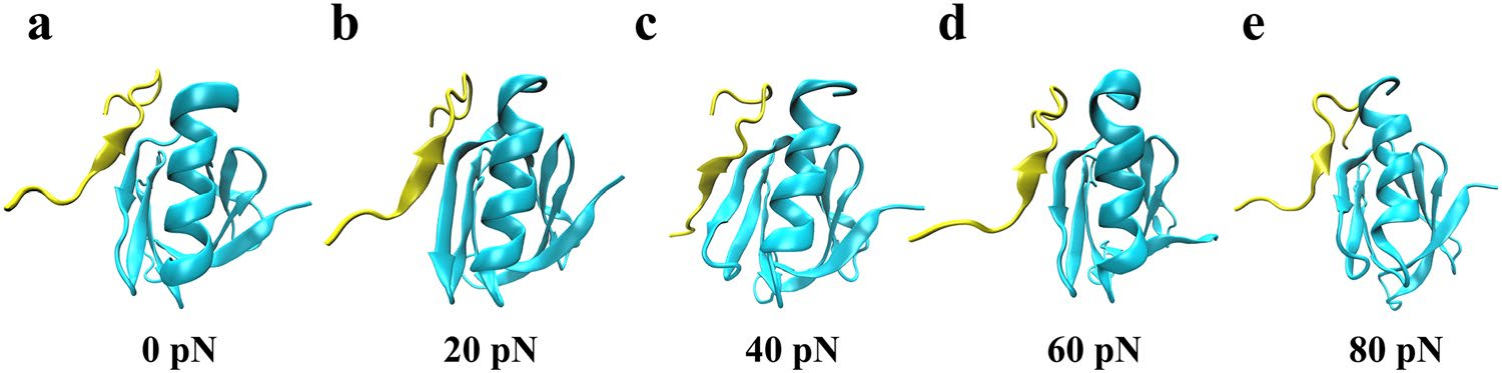
The structure snapshots of the clamped complex under constant tensile forces of 0.0 (**a**), 20 (**b**), 40 (**b**), 60 (**d**) and 80 pN (**e**). At each tensile force ≤ 80pN, unfolding did not occur in simulation, showing a fine mechanical stability and structural conservation of the complex. Each structural snapshot was taken from the samples at simulation time of 40 ns.

**Figure 6:**
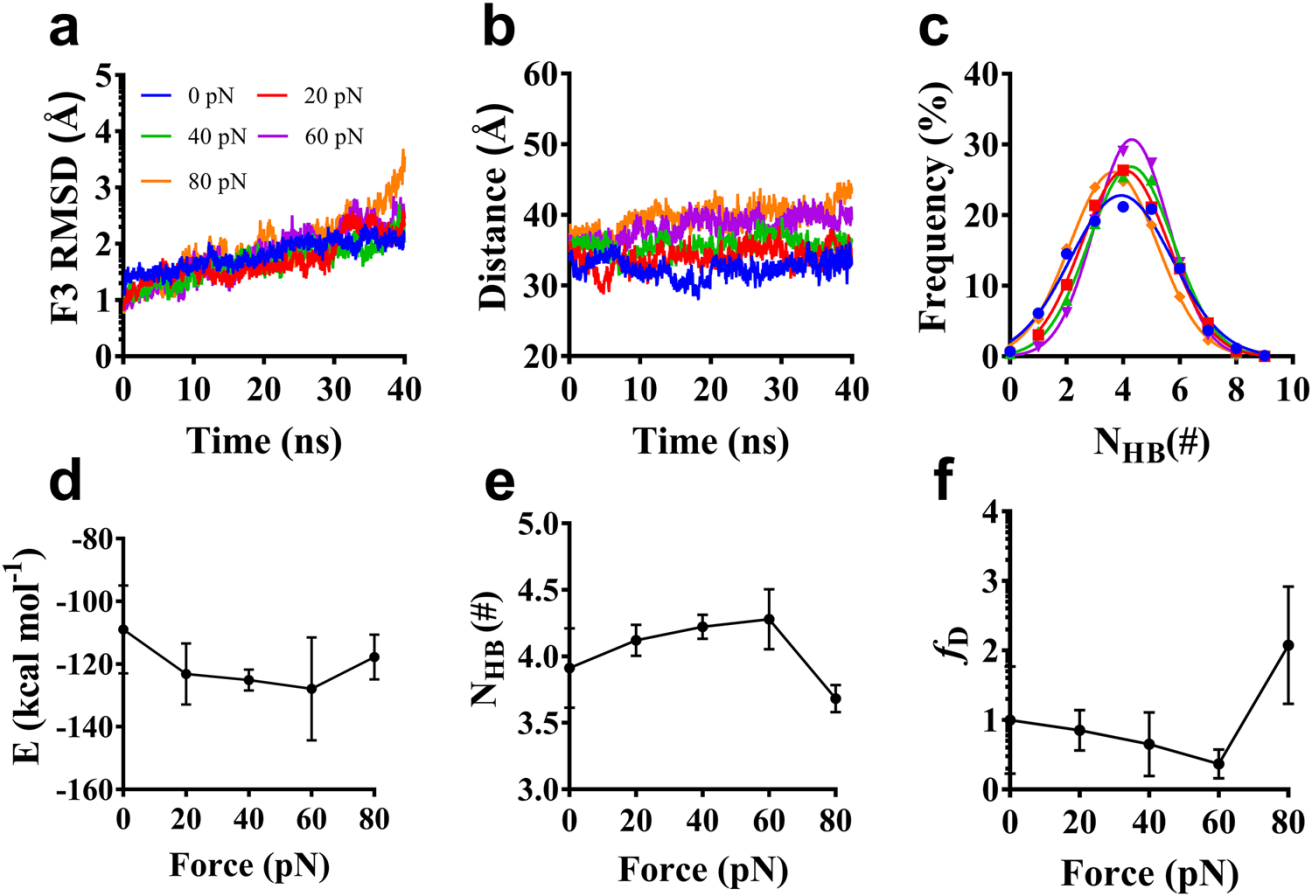
Variation of structural characters and interfacial interaction of complex versus tensile force. All data were from “force-clamp” SMD simulations of 40 ns thrice under tensile forces of zero, 20, 40, 60 and 80 pN. (**a**) The typical time courses of the Cα-RMSD of the ligated Talin F3 domain at various tensile forces. The Cα-RMSD increased slightly (from 1.0 to 3.0 about) with time, possibly coming from structural relaxation of the clamped complex. (**b**) The typical time courses of the distances from the fixed atom to the pulled one loaded with various tensile forces. The mechanical stability of the complex was demonstrated by the distances at a plateau with a slight roughness of 3–5 Å about for each tensile force. (**c**) Gaussian fitting of the N_HB_ frequencies from thrice runs of 40 ns at various tensile forces. The high R^2^ values (> 0.98) for each tensile force implied that the sampled conformational spaces were quasi-perfect. Plots of the mean interaction energy E (**d**), the mean H-bond number N_HB_ (**e**) and the mechano-regulation factor *f*_*D*_ (**f**) against tensile force F, suggested a biphasic force-dependent interaction between Talin and β3 integrin. Pearson correlation coefficients for E, N_HB_, and *f*_*D*_ were -0.36 (*p* > 0.05), 0.42 (*p* < 0.05), and -0.51 (*p* < 0.05) if 0 ≤ F ≤ 60 pN but took 0.27 (*p* > 0.05), − 0.77 (*p* < 0.05) and 0.86 (*p* < 0.01) if 60 pN < F ≤ 80 pN, respectively, statistically demonstrating the force-dependences of E, N_HB_, and *f*_*D*_.

We performed a series of 40 ns “ramp-clamp” SMD simulations thrice on the complex under various constant tensile forces of 0.0, 20, 40, 60 and 80 pN (Materials and Methods) to examine regulation of force on interaction of Talin to β3 integrin. The interaction energies, the buried SASA and the interfacial H-bonds for the complex under constant tensile forces were sampled from the simulations. The results showed that the complex had a high mechano-stability and structural conservation, because of a bit force-induced conformation change of the complex (Fig. 5). The mechanical stability and structural conservation of the complex were reflected also by slight tension-induced increasing of the Cα-RMSD of the complex and distance from pulled- to fixed-atom (Fig. 6a, b). N_HB_, the number of interface H-bonds, obeyed Gaussian distribution, meaning that the conformational space sampled from MD simulation of 40 ns thrice could be regarded as a quasi-perfect one for each given tensile force (Fig. 6c).

Variation of the mean interaction energy (E) and the mean number of the interface H-bonds (N_HB_) over 40 ns for three runs versus tensile force (F) illustrated that E decreased first and then increased with F increasing (Fig. 6d), indicating a biphasic force-dependent energy preference for the stretched β3-integrin/Talin complex; N_HB_ increased first and then decreased with F increasing (Fig. 6e), meaning a transition from force-enhanced to force-weakened linkage between Talin and β3 integrin; as a result, *f*_*D*_, the mechano-regulation factor, decreased first and then increased (Fig. 6f), suggesting a catch-slip bond transition in dissociation of β3 integrin from Talin. A force threshold of 60pN was shared by E, N_HB_ and *f*_*D*_, as it should be. The catch-slip bond phenomenon had been observed by AFM and BFP as well as flow chamber experiments for various adhesive molecule systems, such as LFA-1 with ICAM-1^24^, vWF with ADMAMTS13^39^, α_IIb_β_3_ with fibrinogen^5^ or, and predicted through MD simulations for the molecular systems, such as von Willebrand factor (vWF) with GPIbα^34^ and PSGL-1 with ERM^36^ as well as β3 integrin with Kindin3^40^.

### Force regulation mechanism of the interaction between β3 integrin and Talin F3

**Figure 7:**
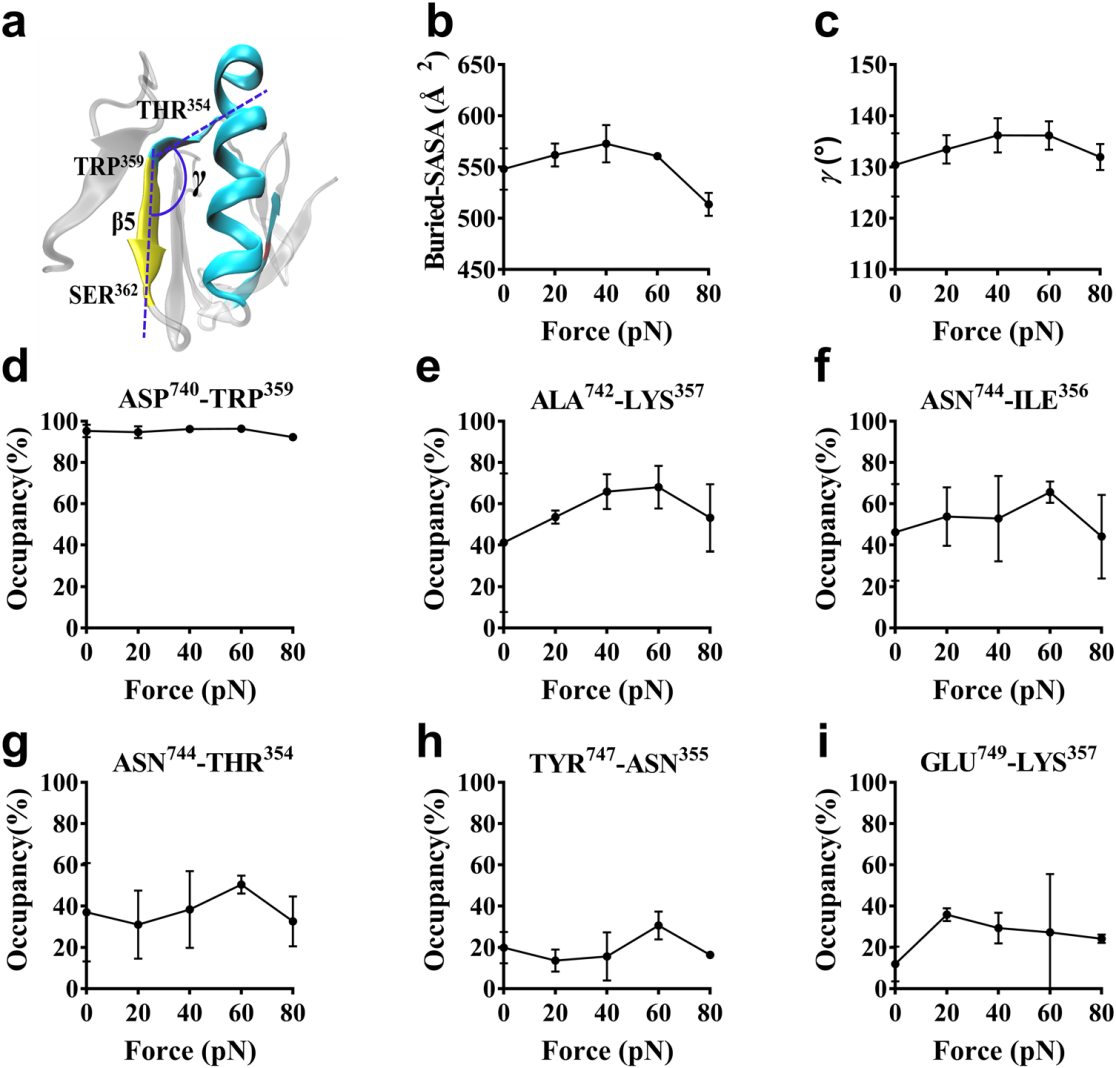
Force regulation mechanism of the interaction between β3 integrin and Talin F3. (**a**) Schematic diagram for β5 strand in Talin F3 domain. The turn angle γ of β5 strand were estimated herein by the cross angle of two lines, which linked two Cα-atoms either in both ALA^360^ and SER^362^ (yellow) or in both TRP^359^ and THR^354^ (cyan). (**b**) Variation of the mean buried SASA versus tensile force. (**c**) Plots of the turn angle γ against tensile force. (**d**) ASP^740^ with TRP^359^, (**e**) ALA^742^ with LYS^357^, (**f**) ASN^744^ with THR^356^, (**g**) ASN^744^ with ILE^354^, (**h**) TYR^747^ with ASN^355^, and (**i**) GLU^749^ with LYS^357^. All data from 40 ns runs thrice were shown in mean ± SD.

External loads would regulate adhesive molecular interactions via mechanical deformations of the ligated molecules, and this mechano-chemical regulation exists widely in various mechanosensitive molecules, including integrins and cytoskeleton-associated proteins^5,20–24^. To uncover the relation of force-induced allostery and unligation of the Talin/β3-integrin complex, we herein analyzed the structural poses sampled from a series of “ramp-clamp” SMD simulation of 40 ns thrice under tensile forces of 0.0, 20, 40, 60 and 80 pN, and examined the force-induced change of the turn angle γ (between the sheet and the loop of β5 strand in the ligated Talin F3 domain), which was calculated by cross angle of two lines, linking two Cα-atoms either in both ALA^360^ and SER^362^ or in both TRP^359^ and THR^354^ (Fig. 7a).

Plot of the turn angle γ against tensile force exhibited that, increasing tensile force made the turn angle γ large first and then small, and the turn point occurred at tensile force of 60 pN too (Fig. 7b). In other words, increasing tensile force could make the β5 strand and its adjacent loop (from TRP^359^ to THR^354^) of the ligated Talin F3 domain spread first and then bent. This force-induced rotation of β5 adjacent loop towards β3 integrin facilitated expanding of complex interface (Fig. 7c), and further enhanced interactions of the binding site residue pairs, such as ALA^742^ with LYS^357^, ASN^744^ with ILE^356^ and with THR^354^ (Fig. 7d-i), leading to increasing of Talin affinity to β3 integrin. So, this force-induced allostery of the clamped Talin should be responsible for the force-enhanced interaction of Talin to β3 integrin, similar to the Kindlin2/β3 integrin complex. By comparing the character of interaction of β3 integrin with Talin and with Kindlin2, the former has better mechanical stability (force threshold, 60 pN) than the latter (20 pN)^40^, although with a smaller interface area than latter. To the best of our knowledge, these MD simulation data provide mechanical proof that Talin plays central role in “inside-out” integrin signaling, while kindlin cooperates with Talin promote the activation of integrin. Meanwhile, Ju et al. works showed that the compression might be sensed and transmitted by the cytoskeleton to this integrin mechno-signalling pathway, and promoted the activation of integrin extracellular domain and plate adhesion to immobilized fibrinogen with the force increasing from 10 to 30 pN, providing the experimental support for this force regulation stability of Talin-integrin linkage^41^.

## Discussion

Talin and β3 integrin, the mechano-transduction protagonists for bi-directional signaling and biophysical linking between extracellular matrix and actin cytoskeleton, are crucial for platelet activation, adhesion, spreading, aggregation and communication with extracellular milieu^5,16,22^. The β3 integrin signaling through Talin were regulated by shear stress in hemodynamics environments^27^. The previous studies showed that the extracellular region of integrins experienced a large-scale extension (more than 13 nm) upon activation^23^ and could withstand 1 ~ 100 pN forces, which were measured via AFM^39^, optical/magnetic tweezers, biomembrance force probe^5^ and molecular tension sensors^42^. Talin head (FERM domain) and rod belonged to modules of integrin signaling and force transduction, respectively^43^. Exposure of binding sites on Talin rod was force-dependent^32^. However, mechanical stability and biophysical connectivity of Talin ligated with β3 integrin remain unclear. We herein examined mechanical regulation on interaction of β3 integrin CT-membrane domain to Talin F3 domain by a series of “ramp-clamp” SMD simulations under various loads, and demonstrated that force-induced allostery might enhance mechanical strength of the complex and affinity of Talin to β3 integrin, based on the force-enhanced interactions of binding site residues and the force-induced spread of β5-strand and adjacent loop on Talin F3 domain.

It is not sure that the present β3 segmental simulation can fully represent the interaction within an intact complex completely. However, this β3 segmental simulation should be a rational for deeply understanding interaction of β3 integrin with Talin, because this β3 segment with Talin F3 domain formed a crucial biophysical knot in the “outside-in” or “inside-out” transmembrane mechano-signaling through the β3 integrin axis, especially in the absence of the crystal structures of intact transmembrane β3 integrin bound with or without Talin^5,44^. The MD simulations showed that, resistance to conformation change of the complex was strong for its high conformational conservation under loads (more than 80 pN), meaning that the complex of Talin with β3 integrin should be a better force transduction module in β3 integrin signaling; and the complex of Talin with β3 integrin had a high mechanical strength for its rupture forces larger than 150 pN, which might be required to prevent the complex from mechanical damage especially in pathological high blood flow shear stresses. It was also from this requirement that, as the early event of breakage of the complex under loads, force-induced deformation of the hydrophobic pocket in F3 domain made the mechanical strength of the complex increased. The conformation changes in a manner to protect the integrin-Talin structure from mechanical stimulus would facilitate mechano-signalling through integrin axis, platelet adhesion and thrombosis^41^. The loop tail of β5 strand in Talin F3 domain would extend through sensing mechanical force (≤60 pN; Fig. 7a, c). This force-induced deformation of β5 strand enhanced binding site residue interactions, enlarged interface of the complex, and let to decreasing of possibility of β3 integrin dissociation from Talin F3 domain, while the transition for these processes would occur at threshold force of 60 pN, showing a force-enhanced biophysical connection of the complex and a “slip-catch bond transition mechanism in interaction between of Talin and β3 integrin under loads. This slip-catch bond transition also had been observed in the interaction of the integrin with its extracellular ligand fibrinogen or fibronectin^5,41^, suggesting a match between interactions of the transmembrane integrin with its intra- and extra-cellular ligands (such Talin and fibrinogen) in mechanical signaling.

The high mechanical strength and the force-enhanced biophysical connection of the complex might be required for not only extension of extracellular region in integrin through breaking the association between α subunit and β subunit near the transmembrane but also transmitting the external forces from ECM or extracellular flow field to Talin rod^16,19^. The force threshold of 60 pN for β3 integrin-Talin was threefold higher than that of β3 integrin-Kindlin2 complex^40^, meaning that Kindlin assistance might be not necessary for the force-enhanced β3 integrin signaling through Talin.

In summary, we here demonstrated a force-regulated interaction between β3 integrin and Talin F3 domain through MD simulations. Our results said that pull-induced dissociation pathway of the complex had two types with or without allostery of hydrophobic pocket in F3 domain, the one with allostery showed a higher mechanical strength than other one, meaning that allostery could prevent the complex from mechanical damage or break. The loop near β5 strand in F3 domain of Talin was force-sensitive, could extend to a conformation in favor to binding with β3 integrin, and might be responsible for the mechanical stability of the complex, the catch-slip bond transition in interaction between β3 integrin and Talin F3 domain, and the stable β3 integrin signaling through Talin. The binding site residue pairs, including ASP^740^-TRP^359^, ALA^742^-LYS^357^, ASN^744^-ILE^356^, ASN^744^-THR^354^ and TYR^747^-ASN^355^ as well as GLU^749^-LYS^357^, were crucial in the force-enhanced β3 integrin signaling through Talin. These results in this work provided a novel insight into β3 integrin activation and signaling through Talin in plate activation, adhesion, spreading and aggregation under blood flows and should be useful for novel drug design and the treatment of related diseases.

## Materials and methods

### System setup

Two software packages, the NAMD 2.13 for molecular dynamics simulation and the Visual Molecular Dynamics (VMD) 1.9.2 for visualization and modeling, were used herein. The crystal structure of complex of Talin/β3 integrin tail came from the PDB database (Protein Data Bank code 1MK7), being composed of the F3 domain of Talin (residue 309–400) and the membrane proximal region of β3 integrin tail (residue 739–749). The terminal patches ACE and CT3 were added to the N-terminal and C-terminal of both integrin β3 tail and Talin F3 domain, respectively, to mimic the continuation of the protein chain^35^. The complex then was soaked with TIP3P water molecules in a rectangular box (83.6 Å × 86.1 Å × 98.4 Å) with walls at least 25 Å away from any protein atom. The system was neutralized with 150 mM Na^+^ and Cl^−^ to mimic the actual physiological environment, and consisted of 67,003 atoms.

### Molecular dynamics simulations

MD simulations were performed with periodical boundary condition and 2 fs timestep as well as the CHARMM27 all-atom force field^36^, along with cMAP correction for backbone, particle mesh Ewald (PME) algorithm for electrostatic interaction, a 12 Å cut off for electrostatic and van der Waals interaction. The system was energy-minimized firstly for 15,000 steps with heavy or non-hydrogen protein atoms being fixed, and then for another 15,000 steps with all atoms free. The energy-minimized systems were heated gradually from 0 to 310 K in 0.1 ns first and then equilibrated thrice for 40 ns with pressure and temperature control. The temperature was held at 310 K using Langevin dynamics, and the pressure was held at 1 atmosphere by the Langevin piston method. The equilibrated structure was used as the initial conformation for the subsequent SMD simulations.

The so-called “ramp-clamp” SMD simulations, a force-clamp MD simulation followed a force-ramp one, were run to examine the regulation of tension on interaction of Talin F3 with β3 integrin. The N-terminal Cα atom (at 736) of β3-tail was fixed, and the N-terminal Cα atom (at 309) of Talin F3 domain was steered along pulling di-rection from the steered atom to the fixed one. The three residues 736 to 738 of the N terminal of β3 integrin in the crystal structure are His tags for protein purification^33^. The virtual spring, connecting the dummy atom and the steered atom, had a spring constant of 13.9 pN/Å. The complex was pulled over 40 ns thrice with the time step of 2 fs and a constant velocity of 3 Å/ns, at which the secondary structure conservation of the complex remained in the pull-induced dissociation of β3 integrin from Talin F3 (Video S1). Once the tensile force f arrived at a given value, such as 20, 40, 60 or 80 pN, the SMD simulation was transform from the force-clamp run mode to a force-ramp one, at which the complex was stretched with the given constant tensile force for the followed 40 ns. Each event of hydrogen bonding under stretching were recorded to examine the involved residues and their functions.

### Data analysis

All analyses were performed with VMD tools. We measured the Cα root mean square deviation (RMSD) and the solvent accessible surface area (SASA) (with a 1.4 Å probe radius) to characterize the conformational change and the hydrophobic core exposure, respectively. We introduced Buried-SASA, the half of the together two parts of β3 and F3 SASA minus whole complex SASA, to characterize the area of the binding surface. A hydrogen bonding event occurred once the donor–acceptor distance and the donor-hydrogen-acceptor angle were less than 3.5 Å and 30°, respectively. A salt bridge was defined if the distance between any of the oxygen atoms of acidic residues (Asp or Glu) and the nitrogen atoms of basic residues (Lys or Arg) must be within 4 Å. An occupancy of a H-bond or a salt bridge was evaluated by the percentage of bond survival time in simulation period. The interaction energy, consisting of van der Waals energy and electrostatic energy, were calculated through the Namdenergy plugin in VMD. As a reflection of the receptor-ligand binding affinity, the rupture force was read from the maximum of the force spectrum in a force-ramp MD simulation with constant pulling velocity. All visual inspections and molecular images were completed by using VMD 1.9.2.

To estimate the residue-residue interactions across binding site through H-bonding, we herein introduced ${p}_{ij}$, the probability of the *i*th ligand residue binding with the *j*th receptor residue, which was defined by

1 $${p}_{ij}=1-\prod_{l=1}^{{M}_{ij}}\left(1-{\omega }_{ij,l}\right), i=\mathrm{1,2},\dots ,{M}_{L};j=\mathrm{1,2},\dots ,{M}_{R}; l=\mathrm{0,1},\dots ,{M}_{ij}$$

where, the formation or breakage of each hydrogen bond on binding site was assumed to be an independent event not related to other bonds, ${\omega }_{ij,l}$ is the survival ratio of the *l*th H-bond between the *i*th ligand residue and the *j*th receptor residue. ${M}_{L}\left(\ge 1\right)$ and ${M}_{R}(\ge 1)$ are respectively the total numbers of ligand and receptor residues involved in binding, and ${M}_{ij}(\ge 0)$ expresses the numbers of H-bonds between the *i*th ligand residue and the *j*th receptor residue. And, ${P}_{j,L}$ (the probabilities of the *j*th ligand residue binding to the receptor) and ${P}_{j,R}$ (the probabilities of the *j*th receptor residue binding to the ligand) were calculated, respectively, approximately by

2 $${P}_{j,L}=1-\prod_{i=1}^{{M}_{R}}\left(1-{p}_{ji}\right) \,\,\mathrm{and } \,\,{P}_{j,R}=1-\prod_{i=1}^{{M}_{L}}\left(1-{p}_{ij}\right)$$

Furthermore, ${P}_{D}$, the dissociation of ligand from receptor, could be estimated by

3 $${P}_{D}=\prod_{j=1}^{{M}_{L}}\left(1-{P}_{j,L}\right)= \prod_{j=1}^{{M}_{R}}\left(1-{P}_{j,R}\right)$$

However, there would be a significant gap between the results from MD simulation and the data measured with single-molecular tools, such as atomic force microscopy (AFM), optical and magnetic tweezers, coming from effects of timescale on predicting ligand-receptor interaction with a timescale of about 0.01–1.00 s by MD simulation of about 100 ns. It was a great challenge to overcome the barrier of the effect of timescale difference of 5–7 order of magnitudes led. We herein introduced ${f}_{D}$, the mechano-regulation factor, which was ratio of ${P}_{D}$ at tensile force of ${f}_{0}$ and of ${P}_{D}$ at zero tensile force, that is

4 $${f}_{D}={P}_{D}{|}_{f={f}_{0}}/{P}_{D}{|}_{f=0}$$

where, *f* expressed the tensile force on the complex. With the assumption of the geometrical the timescale effect on complex dissociation ${P}_{D}$, it was expected that ${f}_{D}$, the mechano-regulation factor or the normalized complex dissociation, should be comparable with experiment data.

All computational simulations were performed in triplicate and results are presented as mean ± standard deviation. Pearson correlation analysis was performed to test the correlation between two different variables. Differences between groups was assayed by a two-tailed student t test; * means *p* < 0.05, ** means *p* < 0.01, *** means *p* < 0.005.

## Supplementary Information

Supplementary Information 1.

Supplementary Video S1a.

Supplementary Video S1b.
