# Supplementary material for: Force-enhanced biophysical connectivity of platelet β3 integrin signaling through Talin is predicted by steered molecular dynamics simulations
Source: Sci Rep. 2022 Mar 17;12:4605. doi: 10.1038/s41598-022-08554-w (PMC8931153; doi:10.1038/s41598-022-08554-w)
Supplement: Supplementary file 1 — Supplementary Information 1. [file 41598_2022_8554_MOESM1_ESM.docx]

# Force-enhanced Biophysical Connectivity of Platelet β3 Integrin Signaling Through Talin is Predicted by Steered Molecular Dynamics Simulations

**Shuixiu Su^1^, Yingchen Ling^1^, Zhiquan Xie^2^, Ying Fang ^1*^, Jianhua Wu^1*^**

**Supplemental Materials**


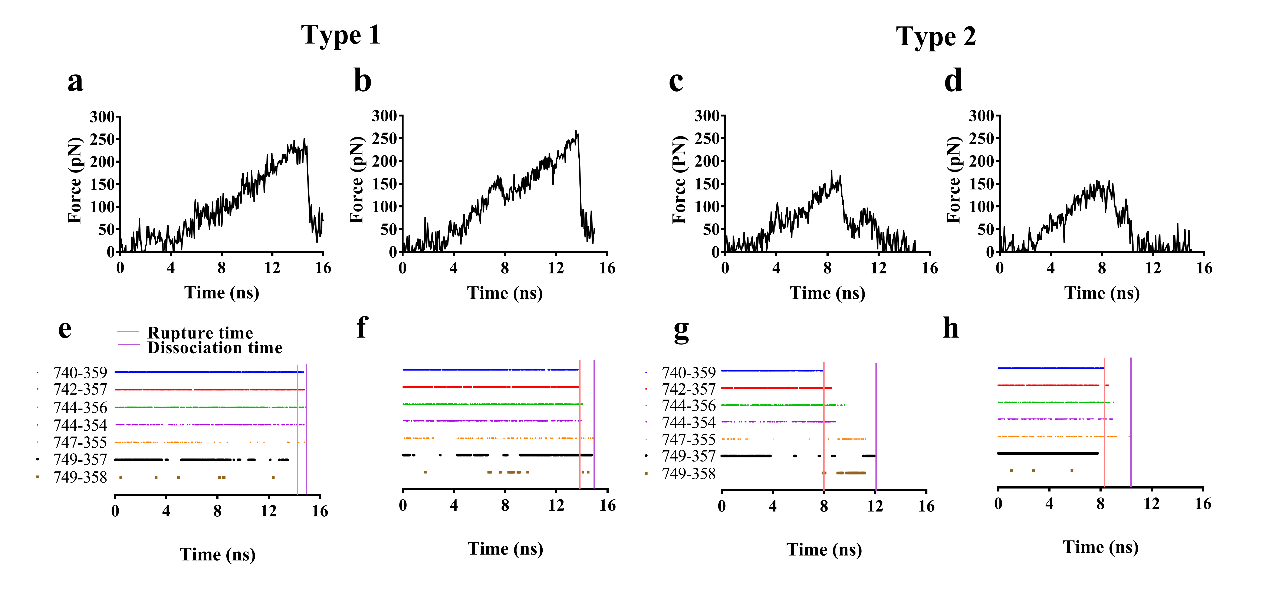


**Figure S1.** The time curves of loading force for another two runs on complex for Type1 (a, b) and Type2 (c, d). The survival patterns of H-bonds across complex interface forType1 (e, f) and Type2 (g, h) another two runs.


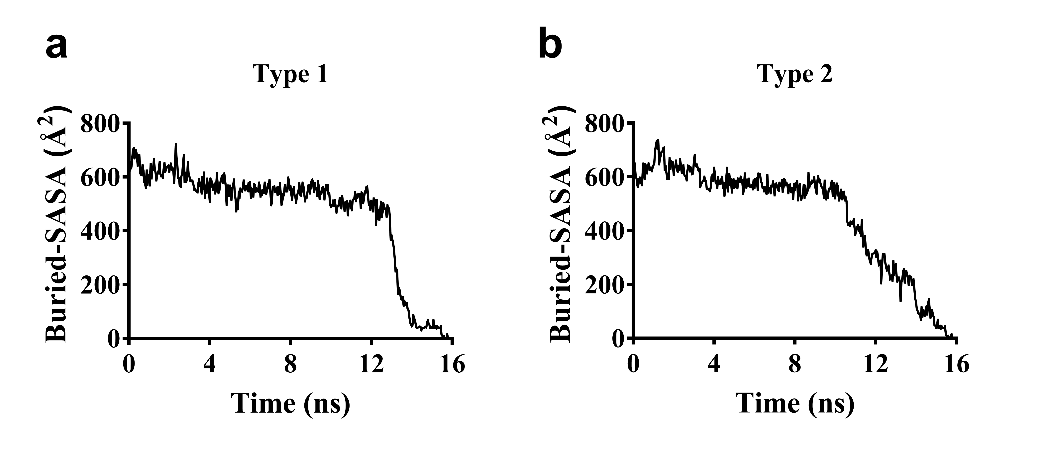


Figure S2. The time-curves of Buried-SASA in type 1 (a) and 2 (b), We introduced Buried-SASA, the half of the together two parts of β3 and F3 SASA minus whole complex SASA, to characterize the area of the binding surface.

Video S1. The movies were shown a typical dissociation process of β3/F3 complex in Type1 (a) and Type2 (b) (The yellow color is integrin β3 tail, and the cyan color is Talin F3, and the purple sphere is the fixed atom, and the blue sphere is the steered atom).
